# Quantification of perineural invasion on prostate biopsy improves risk stratification in biopsy Grade Group 2–3 cancer

**DOI:** 10.1002/bco2.70196

**Published:** 2026-03-31

**Authors:** Yuki Teramoto, Ying Wang, Hiroshi Miyamoto

**Affiliations:** ^1^ Department of Pathology & Laboratory Medicine University of Rochester Medical Center Rochester New York USA; ^2^ Department of Urology University of Rochester Medical Center Rochester New York USA; ^3^ James P. Wilmot Cancer Institute University of Rochester Medical Center Rochester New York USA

**Keywords:** needle biopsy, perineural invasion, prognosis, prostate cancer, radical prostatectomy

## Abstract

**Objective:**

Perineural invasion (PNI) detected on prostate needle biopsy is a well‐established indicator of adverse pathology, while the prognostic role of PNI quantification has recently been suggested. We herein aimed to determine whether PNI quantification could universally stratify risks by separately assessing subgroups of patients based on biopsy Grade Group (GG).

**Methods:**

We quantified actual PNI foci in the entire systematic sextant biopsy specimens from 840 men, including 580 (69.0%) exhibiting no PNI and evaluated long‐term oncologic outcomes following radical prostatectomy.

**Results:**

PNI was detected in 1 (n = 177; 21.1%), 2 (n = 48; 5.7%) or 3–6 (n = 35; 4.2%) of 6 biopsy sites/parts, while 1 (n = 156; 18.6%), 2 (n = 53; 6.3%) or 3–10 (n = 51; 6.1%) PNI foci were present on each biopsy. In the entire cohort, we confirmed a significantly higher risk of biochemical recurrence in patients with PNI (vs. no PNI; *P* < 0.001), multi‐site PNI (vs. single‐site PNI; *P* < 0.001) or multifocal PNI (vs. unifocal PNI; *P* < 0.001). In subgroup analyses, significant differences in the risk of postoperative recurrence between the absence vs. presence of PNI were observed only in patients with GG3 or GG4 cancer. In contrast, the prognostic distinction between single‐site vs. multi‐site PNI or unifocal vs. multifocal PNI was evident in patients with GG2 or GG3 cancer, but not in those with GG1 or GG4–5 cancer. In multivariable analysis, multifocal PNI, but not multi‐site PNI, was independently associated with worse recurrence‐free survival in the GG2 (hazard ratio 5.866, *P* = 0.004) and GG3 (hazard ratio 2.716, *P* = 0.021) groups. Meanwhile, PNI on prostatectomy was confirmed in 98.1% of biopsy PNI‐positive cases but was still detected in 72.2% of biopsy PNI‐negative cases.

**Conclusion:**

Quantification of actual PNI foci across all systematic biopsy sites may provide valuable prognostic information, particularly in patients with GG2–3 cancer. Notably, the mere presence of biopsy PNI may not universally predict poorer outcomes.

## 1 INTRODUCTION

Prostate cancer continues to rank among the most prevalent malignancies globally in men, with a notable increase in cancer‐related deaths in recent years.^1^, ^2^ Definitive therapy, such as radical prostatectomy, offers curative potential for most patients with localized disease. However, a substantial number of these patients subsequently develop recurrent disease,^3^, ^4^ highlighting the importance of accurate risk stratification at not only post‐treatment but also initial cancer diagnosis.

Histopathologic findings of prostate cancer on needle core biopsy often provide vital information for optimal patient management. In addition to the well‐established Gleason score/Grade Group (GG), perineural invasion (PNI) by prostate cancer has reliably been associated with adverse pathologic features,^5^, ^6^ particularly extraprostatic extension as a major route.^7^ Indeed, the detection of PNI on prostate biopsy has been recognized as an independent prognosticator in patients undergoing radical prostatectomy or radiotherapy.^6^, ^8^, ^9^, ^10^

Currently, pathologists do not routinely count the number of PNI foci in prostate cancer specimens, while a subset of them even report the presence of PNI as a case‐level summary (e.g. ‘PNI identified in this case’^11^) on biopsy without specifying the site(s)/part(s) exhibiting PNI. Recently, we assessed systematic prostate biopsies and found that PNI involving multiple biopsy sites (vs. a single biopsy site)^12^ or multiple foci (vs. a single focus), even those detected in a single biopsy site,^13^ was strongly associated with worse histopathologic features on corresponding radical prostatectomy and poorer oncologic outcomes as independent predictors. Interestingly, the risks of postoperative recurrence were not significantly different between cases with no PNI vs. only a single focus of PNI,^13^ nor between those with PNI in 2 vs. ≥3 biopsy sites^12^ or 2 vs. ≥3 foci.^13^ The present study then aimed to determine whether the prognostic impact of PNI quantification on prostate biopsy was universally seen in subgroups of patients stratified by biopsy GG.

## 2 MATERIALS AND METHODS

### 2.1 Study design and patients

Following the approval by the Institutional Review Board, we retrospectively assessed consecutive patients undergoing systematic prostate needle core biopsy (six sites—right‐apex/right‐mid/right‐base/left‐apex/left‐mid/left‐base; typically two cores/site) with or without concurrent MRI‐targeted biopsy, followed by robot‐assisted radical prostatectomy, both performed at our institution between 2010 and 2016. From our surgical pathology database, we identified 840 men with conventional prostatic adenocarcinoma after excluding cases exhibiting prostate cancer on targeted biopsy (whose prognostic impact had been suggested to be greater than that on systematic biopsy^14^) or undergoing neoadjuvant therapy before radical prostatectomy, and those where the histology slides were unavailable for re‐review.

### 2.2 Data collection and analysis

We measured the summed tumour length (doubled in a small subset of cases with only 1 core or averaged for 2 cores in those with ≥3 cores at each biopsy site). In the entire biopsy specimen, we also counted PNI, defined as the presence of prostate cancer directly adjacent to a nerve but not necessarily encircled completely by tumour glands/cells,^15^, ^16^ and separately recorded the numbers of biopsy sites involving PNI and actual PNI foci across all biopsy sites. Gleason score/GG on biopsy, as well as radical prostatectomy where the entire prostate had primarily been submitted for histological assessment, was also re‐evaluated in accordance with recent recommendations by the Genitourinary Pathology Society^17^ and International Society of Urological Pathology,^18^ while intraductal carcinoma of the prostate was incorporated into grade assignments, as advocated by the latter. The highest score among multiple cores in a single systematic biopsy site was recorded. Then, the highest score across all biopsy sites in each case, as well as the score assigned to a single dominant nodule in each prostatectomy specimen, was used for final analysis. Additionally, in radical prostatectomy specimens exhibiting GG2 (n = 19) or GG3 (n = 30) cancer, <5% of a minor tertiary pattern 5 was not incorporated into the analysis. We also collected other histopathologic findings, including pT and pN staging categories, surgical margin status and estimated tumour volume, from pathology reports and confirmed by re‐review.

Clinical data, including age at biopsy, preoperative prostate‐specific antigen (PSA) value and follow‐up outcomes, were retrieved from the hospital's electronic medical record system (last accessed in November 2025). Biochemical recurrence (BCR) was defined as a single PSA level of ≥0.2 ng/ml, except in patients receiving hormonal therapy (n = 22), radiotherapy (n = 42), or both (n = 27) immediately post‐surgery due to potential residual disease or high‐risk disease, where BCR was defined as a PSA rise of ≥2 ng/ml over nadir^19^ or the commencement of salvage therapy.

Statistical analyses included the Student's *t*‐test for continuous variables and the chi‐square test or Fisher's exact test for non‐continuous variables. Kaplan–Meier analysis was performed to estimate BCR‐free survival (censored at the last available PSA test), with group comparisons via the log‐rank test. The Cox proportional hazards models were applied to assess BCR risks. All statistical analyses were conducted using EZR software (R version 4.0.2^20^) or Prism version 10.6.1 (GraphPad Software), with significance set at *P* < 0.05.

## 3 RESULTS

We examined, in a blinded manner, a total of 840 sets of sextant prostate biopsy and corresponding radical prostatectomy. Table S1 summarizes the clinicopathologic characteristics of these patients, including 580 (69.0%) cases exhibiting no PNI on their biopsies. PNI was thus detected in 1 (n = 177; 21.1%), 2 (n = 48; 5.7%), 3 (n = 18; 2.1%), 4 (n = 10; 1.2%), 5 (n = 3; 0.4%) or 6 (n = 4; 0.5%) of 6 biopsy sites/parts, while 1 (n = 156; 18.6%), 2 (n = 53; 6.3%), 3 (n = 21; 2.5%), 4 (n = 13; 1.5%) or 5–10 (n = 17; 2.0%) PNI foci were present on each entire biopsy. We then assessed the clinical impact of: 1) the absence vs. presence of PNI; 2) the number of biopsy sites involving PNI; and 3) the total number of actual PNI foci across all biopsy sites, in not only the entire cohort of patients but also subgroups of patients stratified by biopsy GG. Table 1 summarizes univariate survival analysis data in the entire cohort of patients, as well as subgrouped patients (for subsections 3.1, 3.3).

**TABLE 1:** Summary of the prognostic significance of perineural invasion (PNI) on biopsy (Bx) in the entire cohort and subgroups.

PNI	Univariate analysis for recurrence‐free survival							
	Log‐rank test	All	Bx GG1	Bx GG2	Bx GG3	Bx GG4	Bx GG5	Bx GG4–5
		n = 840	n = 282	n = 293	n = 140	n = 98	n = 27	n = 125
(−) vs. (+)	HR 95% CI *P*	**3.074** **2.197–4.301** **<0.001**	1.174 0.247–5.582 0.830	1.430 0.778–2.629 0.231	2.892 1.659–5.043 <0.001	**3.036** **1.620–5.690** **<0.001**	1.063 0.359–3.142 0.913	2.462 1.443–4.200 0.001
0 vs. 1 Bx site	HR 95% CI *P*	2.361 1.554–3.589 <0.001	0.644 0.122–3.392 0.665	1.098 0.556–2.169 0.784	2.165 1.011–4.636 0.023	3.600 1.601–8.098 <0.001	1.392 0.396–4.892 0.595	3.075 1.547–6.110 <0.001
1 vs. ≥2 Bx sites	HR 95% CI *P*	2.123 1.343–3.356 <0.001	9.195 0.087–971.0 0.056	2.853 0.860–9.463 0.018	1.999 1.003–3.985 0.039	0.665 0.312–1.415 0.299	0.554 0.147–2.093 0.370	0.602 0.313–1.158 0.133
0 vs. 1 focus	HR 95% CI *P*	1.491 0.935–2.377 0.060	0.644 0.122–3.392 0.665	0.771 0.375–1.586 0.502	1.342 0.552–3.264 0.486	2.310 0.810–6.587 0.052	1.359 0.351–5.257 0.643	2.195 0.943–5.112 0.029
1 vs. ≥2 foci	HR 95% CI *P*	**4.434** **2.859–6.876** **<0.001**	9.195 0.087–971.0 0.056	**5.868** **1.839–18.73** **<0.001**	**3.796** **1.934–7.450** **<0.001**	1.644 0.763–3.540 0.231	0.654 0.165–2.589 0.522	1.247 0.637–2.442 0.530

*Note*: Bold numbers: Independent factor in multivariable analysis. Pale grey cells: 0.05 ≤ *P* < 0.1. Grey cells: *P* < 0.05.

### 3.1 Clinical impact of the absence vs. presence of PNI

We first compared cases without versus with PNI on biopsy. The presence of PNI was significantly associated with adverse clinicopathologic features, including higher PSA, higher tumour grade and higher tumour length/volume on biopsy/prostatectomy, higher pT staging category, higher rates of lymph node metastasis and positive surgical margin and higher necessity of adjuvant therapy immediately after radical prostatectomy (Table S2). In the comparisons in each subgroup, significant differences were seen in tumour length on biopsy, pT stage and tumour volume on prostatectomy. In addition, the presence of PNI was strongly associated with the need for adjuvant therapy in the GG3 group, as well as higher GG on prostatectomy and lymph node metastasis in the GG4–5 group.

Univariate survival analysis was then performed to assess the impact of biopsy PNI on the prognosis following radical prostatectomy. As expected, in the entire cohort, patients exhibiting PNI had a significantly higher risk of BCR (*P* < 0.001; Figure S1A). While there were no significant differences in BCR‐free survival between GG1 (*P* = 0.830; Figure S1B), GG2 (*P* = 0.231; Figure S1C) or GG5 (*P* = 0.913; Figure S1F) cancer cases without vs. with PNI, the risk of BCR was significantly higher in GG3 (*P* < 0.001; Figure S1D), GG4 (*P* < 0.001; Figure S1E) or GG4–5 (*P* = 0.001; Figure S1G) cancer cases exhibiting PNI.

To determine whether biopsy PNI was an independent predictor of postoperative BCR, we further performed multivariable analyses adjusting for other established prognostic factors, including PSA, tumour length on biopsy, GG, pT, pN, surgical margin and tumour volume on prostatectomy, using Cox proportional hazards models, in the entire cohort of patients (Table S3) or those with GG3 (Table S4) or GG4 (Table S5) cancer detected on biopsy. The presence of PNI showed significance in the entire cohort [hazard ratio (HR) 1.440, 95% confidence interval (CI) 1.014–2.045, *P* = 0.042] and the GG4 group (HR 2.705, 95% CI 1.186–6.169, *P* = 0.018) for the risk of BCR, as well as marginal significance in the GG3 group (HR 1.918, 95% CI 0.977–3.763, *P* = 0.058).

### 3.2 Clinical impact of the number of biopsy sites with PNI

We next assessed the clinical impact of PNI site/part number, particularly a single biopsy site versus multiple biopsy sites involving PNI. In the entire cohort, multi‐site PNI was significantly associated with all the adverse clinicopathologic features examined, except PSA (Table S6). Significant differences in some of these, such as biopsy tumour length (both), prostatectomy GG (GG2 only), pT stage (both), pN stage (GG4–5 only), prostatectomy tumour volume (GG2 only) and the need for adjuvant therapy (both), between cases with single‐site PNI vs. multi‐site PNI were seen in the GG2 and GG4–5 groups, but not in the GG1 and GG3 groups.

**FIGURE 1:**
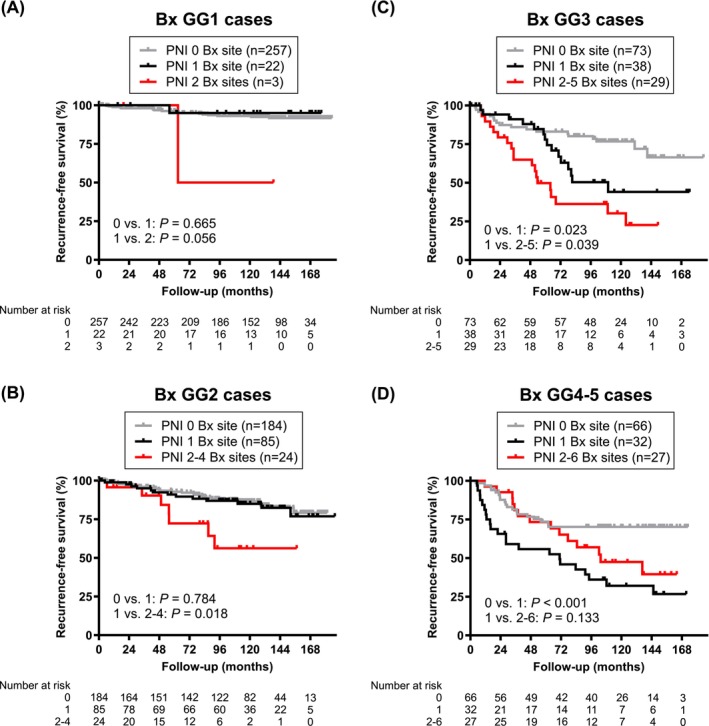
Prognostic significance of the number of PNI sites on biopsy. Kaplan–Meier curves for BCR‐free survival in patients with biopsy GG1 (A), GG2 (B), GG3 (C) or GG4–5 (D) cancer where PNI was detected in 0, 1 or 2–6 biopsy sites. Comparison between 2 groups was made by the log‐rank test. Bx, biopsy.

Univariate analysis in all patients showed significant differences in BCR‐free survival between those with no PNI vs. single‐site PNI (*P* < 0.001), as well as between those with a single site vs. multiple sites of PNI (*P* < 0.001) (Figure S2A/S2B). In subgroup analyses, the prognostic differences between single‐site PNI vs. multi‐site PNI were significant in the GG2 (*P* = 0.018; Figure 1B) and GG3 (*P* = 0.039; Figure 1C) groups, but not in the GG1 (*P* = 0.056; Figure 1A), GG4 (*P* = 0.299; Figure S2C), GG5 (*P* = 0.370; Figure S2D) and GG4–5 (*P* = 0.133; Figure 1D) groups.

In multivariable Cox regression analyses, multiple biopsy sites with PNI (vs. single‐site) did not show significance for the risk of postoperative BCR in the entire cohort of patients (HR 1.007, 95% CI 0.629–1.611, *P* = 0.977; Table S7), GG2 cancer cases (HR 2.173, 95% CI 0.612–7.714, *P* = 0.230; Table S8) and GG3 cancer cases (HR 1.470, 95% CI 0.665–3.251, *P* = 0.341; Table S9).

### 3.3 Clinical impact of the total number of PNI foci

We then assessed the clinical impact of actual PNI foci quantification, particularly unifocal versus multifocal PNI. In the entire cohort, multifocal PNI was significantly associated with all the adverse clinicopathologic features examined, except PSA (Table S10). Significant differences in some of these, such as biopsy tumour length (both), pT stage (both), pN stage (GG4–5 only), prostatectomy tumour volume (GG2 only) and the need for adjuvant therapy (GG2 only), between cases with unifocal PNI vs. multifocal PNI were seen in the GG2 and GG4–5 groups, but not in the GG1 and GG3 groups.

**FIGURE 2:**
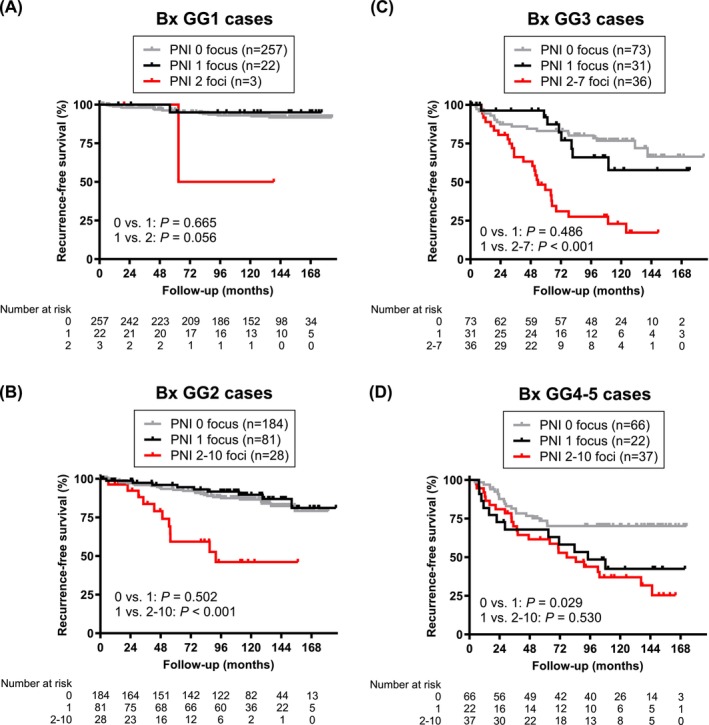
Prognostic significance of the number of PNI foci on biopsy. Kaplan–Meier curves for BCR‐free survival in patients with biopsy GG1 (A), GG2 (B), GG3 (C) or GG4–5 (D) cancer where PNI was detected in 0, 1 or 2–10 foci. Comparison between 2 groups was made by the log‐rank test. Bx, biopsy.

Univariate analysis in all patients showed a significant difference in BCR‐free survival between those with unifocal PNI vs. multifocal PNI (*P* < 0.001; Figure S3A/S3B). Additionally, consistent with our previous observations,^13^ there was no significant prognostic difference between cases with no PNI vs. single PNI (*P* = 0.060), although the difference was significant in the GG4–5 group (*P* = 0.029; see Figure 2D). In subgroup analyses, the prognostic differences between unifocal PNI vs. multifocal PNI were significant in the GG2 (*P* < 0.001; Figure 2B) and GG3 (*P* < 0.001; Figure 2C) groups, but not in the GG1 (*P* = 0.056; Figure 2A), GG4 (*P* = 0.231; Figure S3C), GG5 (*P* = 0.522; Figure S3D) and GG4–5 (*P* = 0.530; Figure 2D) groups.

In multivariable Cox regression analyses, multifocal PNI (vs. unifocal PNI) showed significance for the risk of postoperative BCR in the entire cohort of patients (HR 2.357, 95% CI 1.410–3.942, *P* = 0.001; Table S11), GG2 cancer cases (HR 5.866, 95% CI 1.768–19.46, *P* = 0.004; Table 2) and GG3 cancer cases (HR 2.716, 95% CI 1.159–6.363, *P* = 0.021; Table 3).

**TABLE 2:** Multivariable analysis of prognostic factors, including PNI in a single focus vs. multiple foci on biopsy, in biopsy GG2 cases.

	HR	95% CI	*P*
**PSA**	1.025	0.890–1.181	0.729
**Biopsy tumour length**	1.023	0.986–1.061	0.226
**PNI**			
1 focus	Reference		
≥2 foci	5.866	1.768–19.46	0.004
**Prostatectomy Grade Group**			
1–2	Reference		
3	0.749	0.221–2.546	0.644
4	<0.001	<0.001‐Inf	0.998
5	NA	NA	NA
**pT**			
2	Reference		
3a	3.106	0.921–10.48	0.068
3b	2.100	0.231–19.12	0.510
**Lymph node involvement**	43.624	0.128–102.3	0.450
**Surgical margin**	2.674	0.894–8.000	0.079
**Prostatectomy tumour volume**	1.047	0.979–1.120	0.183

CI, confidence interval; HR, hazard ratio; NA, not available; PNI, perineural invasion; PSA, prostate‐specific antigen.

**TABLE 3:** Multivariable analysis of prognostic factors, including PNI in a single focus vs. multiple foci on biopsy, in biopsy GG3 cases.

	HR	95% CI	*P*
**PSA**	1.011	0.995–1.027	0.170
**Biopsy tumour length**	1.002	0.984–1.021	0.846
**PNI**			
1 focus	Reference		
≥2 foci	2.716	1.159–6.363	0.021
**Prostatectomy Grade Group**			
1–2	Reference		
3	2.130	0.782–5.802	0.139
4	2.681	0.622–11.56	0.186
5	2.800	0.780–10.06	0.115
**pT**			
2	Reference		
3a	3.067	0.386–24.38	0.289
3b	3.169	0.353–28.43	0.303
**Lymph node involvement**	2.243	0.622–8.090	0.217
**Surgical margin**	1.374	0.501–3.764	0.537
**Prostatectomy tumour volume**	1.029	0.966–1.096	0.375

CI, confidence interval; HR, hazard ratio; PNI, perineural invasion; PSA, prostate‐specific antigen.

### 3.4 Clinical impact of PNI quantification in patients not undergoing adjuvant therapy before recurrence

The criteria for BCR in the present study were distinct between patients without versus with adjuvant therapy immediately after prostatectomy. Therefore, we additionally assessed only the former patients by excluding 91 with immediate postoperative adjuvant therapy.

Table 4 summarizes survival analysis data in these 749 patients and their subgroups stratified by biopsy GG. Similar results were obtained in all analyses except univariate survival data for single‐site PNI vs. multi‐site PNI in GG2 or GG3 cancer cases and 0 vs. 1 focus of PNI in GG4–5 cancer cases (i.e. elimination of statistical significance). Thus, in multivariable analyses, multifocal PNI (vs. unifocal PNI) remained significantly predictive of BCR in all patients (HR 2.384, 95% CI 1.225–4.642, *P* = 0.011), as well as in GG2 (HR 6.458, 95% CI 1.664–25.07, *P* = 0.007) or GG3 (HR 4.213, 95% CI 1.201–14.78, *P* = 0.025) cancer cases.

**TABLE 4:** Summary of the prognostic significance of perineural invasion (PNI) on biopsy (Bx) in patients not undergoing immediate postoperative adjuvant therapy.

PNI	Univariate analysis for recurrence‐free survival							
	Log‐rank test	All	Bx GG1	Bx GG2	Bx GG3	Bx GG4	Bx GG5	Bx GG4–5
		n = 749	n = 277	n = 272	n = 113	n = 73	n = 14	n = 87
(−) vs. (+)	HR	**2.973**	1.276	1.440	2.996	**3.280**	1.072	2.676
	95% CI	**1.988–4.446**	0.253–6.428	0.713–2.909	1.572–5.710	**1.477–7.288**	0.268–4.291	1.337–5.356
	*P*	**<0.001**	0.744	0.288	<0.001	**0.002**	0.921	0.004
	HR	2.313	0.703	1.202	2.228	4.125	1.170	3.313
	95% CI	1.430–3.743	0.125–3.969	0.560–2.579	0.880–5.642	1.590–10.70	0.256–5.343	1.460–7.513
	*P*	<0.001	0.731	0.627	0.039	<0.001	0.836	<0.001
0 vs. 1 Bx site								
1 vs. ≥2 Bx sites	HR	2.290	8.692	2.551	1.869	0.502	0.679	0.519
	95% CI	1.265–4.143	0.091–827.7	0.552–11.80	0.849–4.113	0.189–1.337	0.084–5.468	0.215–1.256
	*P*	<0.001	0.065	0.092	0.116	0.215	0.735	0.190
0 vs. 1 focus	HR	1.359	0.703	0.880	1.324	2.258	1.129	2.029
	95% CI	0.795–2.324	0.125–3.969	0.393–1.971	0.446–3.931	0.606–8.419	0.199–6.398	0.692–5.956
	*P*	0.220	0.731	0.761	0.580	0.133	0.888	0.123
1 vs. ≥2 foci	HR	**5.544**	8.692	**5.567**	**3.846**	1.980	1.069	1.715
	95% CI	**3.168–9.700**	0.091–827.7	**1.271–24.38**	**1.756–8.425**	0.762–5.147	0.150–7.597	0.727–4.044
	*P*	**<0.001**	0.065	**<0.001**	**0.003**	0.189	0.946	0.236

*Note*: Bold numbers: Independent factor in multivariable analysis. Pale grey cells: 0.05 ≤ *P* < 0.1. Grey cells: *P* < 0.05.

## 4 DISCUSSION

It has been well documented that detection of PNI on prostate biopsy is a strong indicator of more aggressive disease, especially extraprostatic extension, and resultant poorer oncologic outcome.^5^, ^6^, ^8^, ^9^, ^10^, ^13^ Various studies have indeed indicated the independent value of biopsy PNI to predict BCR following definitive therapy. More recently, we have suggested the prognostic significance of PNI quantification on systematic biopsy by demonstrating significant differences in the risk of postoperative BCR between cases with PNI in a single biopsy site vs. multiple biopsy sites,^12^ as well as between those with a single focus of PNI vs. multiple foci of PNI.^13^ In the latter study,^13^ the prognosis was found to be significantly worse in cases with multifocal PNI detected only in a single biopsy site than in those with unifocal PNI. In the present study, we further assessed the clinical significance of: 1) the absence vs. presence of PNI; 2) a single biopsy site vs. multiple biopsy sites involving PNI; and 3) a single focus vs. multiple foci of PNI, separately in subgroups of patients stratified by biopsy GG.

We first confirmed the prognostic values of the presence of PNI, PNI in multiple biopsy sites, and multifocal PNI in the entire cohort of patients in univariate analyses. After subgroup analyses, the prognostic value of the absence vs. presence of PNI was affirmed only in the GG3, GG4 and GG4–5 groups. However, multivariable analysis identified its independent value only in GG4 cancer cases, but not in GG3 (or GG4–5) cancer cases with which other adverse prognostic factors might be more closely associated. Similarly, the significant differences in the prognosis between single‐site PNI vs. multi‐site PNI or unifocal PNI vs. multifocal PNI were both affirmed only in the GG2 and GG3 groups. In both of these subgroups, multifocal PNI was found to be an independent prognosticator, whereas multivariable analysis failed to show the prognostic significance of multi‐site PNI. Thus, quantification of actual PNI foci on each entire biopsy may particularly be useful for more accurately predicting the postoperative risk of disease recurrence in men initially diagnosed with GG2 or GG3 prostate cancer.

The clinical impact of PNI on radical prostatectomy remains controversial.^21^ Specifically, several studies have failed to demonstrate the prognostic significance of PNI detection or its extent or quantity in prostatectomy specimens.^21^, ^22^, ^23^, ^24^ We further evaluated the status of PNI in radical prostatectomy specimens where PNI was identified in a total of 674 (80.2%) cases. We then assessed the concordance of PNI status between biopsy and prostatectomy (Table S12). PNI was confirmed at prostatectomy in the vast majority [i.e. 255 (98.1%) of 260] of biopsy PNI‐positive cases, while PNI was also detected in 419 (72.2%) of 580 biopsy PNI‐negative cases. Notably, in our cohort, the presence of PNI on prostatectomy was associated with a significantly higher risk of BCR, compared with its absence (HR 5.049, 95% CI 2.580–9.880, *P* < 0.001; Figure S4).

The detection of clinically significant prostate cancer has been noticeably improved by the introduction of the Prostate Imaging Reporting and Data System (PI‐RADS) classification on multiparametric MRI and biopsy of the target lesion.^25^, ^26^ As we previously demonstrated,^6^, ^27^, ^28^ the presence of PNI on targeted biopsy was associated with a significantly higher risk of postoperative BCR. More strikingly, patients with PNI detected on both systematic biopsy and targeted biopsy had a significantly higher risk of BCR than those with PNI only on systematic biopsy even when the prognosis was compared between cases with PNI in one of systematic biopsy sites plus the target lesion (i.e. total 2 sites) vs. two of systematic biopsy sites.^27^ Moreover, we previously demonstrated that the detection of prostate cancer on targeted biopsy, in addition to or instead of systematic biopsy, was associated with significantly worse postoperative outcomes.^14^ The clinical impact of not only PNI but also cancer detection on targeted biopsy may thus be greater than that on systematic biopsy. We therefore used only systematic biopsy data, while excluding cases with prostate cancer detected on targeted biopsy in the present study, to impartially assess the prognostic value of PNI quantification.

The present study has potential limitations that warrant consideration. Firstly, its retrospective design and single‐institution setting may restrict the broader applicability of our findings. Secondly, our analysis was limited to radical prostatectomy cases, and the clinical significance of PNI quantification in non‐surgical populations could not be evaluated. Thirdly, non‐significant data in the GG1 group might be due to the relatively small sample size of the PNI cases, particularly only three exhibiting multi‐site/multifocal PNI. Fourthly, the assessment of actual PNI foci detected in different cores in one of traditional sextant 12‐core systematic biopsy sites might have possibly introduced an ‘enrichment bias’ when the same lesion was biopsied multiple times, although the prognostic distinction between a single focus vs. multiple foci of PNI even within a single biopsy site/part (i.e. possibly a single lesion) was evident in our previous study.^13^ Moreover, sextant biopsy may no longer reflect the standard practice. Fifthly, we excluded cases in which prostate cancer had been detected on targeted biopsy to maintain consistency in our cohort of 2010–2016 cases (i.e. targeted biopsy started in 2014 in our institution^14^), while our previous observations suggested a greater impact of cancer itself^14^ or PNI^27^ detected on targeted biopsy than that on systematic biopsy, as described above. Sixthly, we did not consider the degree of PNI (e.g. encircled completely vs. incompletely by cancer) or the size of PNI or cancer at PNI focus, although our recent studies suggested limited clinical impact of these factors.^16^, ^29^ Seventhly, while the interobserver variability in the diagnosis of PNI had been noted,^30^ no use of immunostaining for nerve markers in virtually all biopsy specimens might have resulted in missing actual PNI foci and consequently generating a bias. Finally, we have never assessed the concordance of PNI counts in biopsy and corresponding prostatectomy specimens, while the prognostic significance of prostatectomy PNI remains debatable.

## 5 CONCLUSIONS

The presence of PNI on systematic needle core biopsy of the prostate was found to be an independent predictor of poorer postoperative oncologic outcomes only in patients diagnosed with GG4 prostate cancer. Similarly, biopsy PNI detected in multiple biopsy sites (vs. single site) or multiple actual foci (vs. single focus) accurately stratified the risk of postoperative BCR in patients with GG2‐3 prostate cancer, although multi‐site PNI was not an independent prognosticator. The distinction of single vs. multifocal PNI on biopsy may thus improve the postoperative risk stratification of prostate cancer and possibly the decision of treatment options particularly in men initially diagnosed with GG2 or GG3 disease. Meanwhile, as demonstrated in GG1‐2 or GG5 cancer cases, the presence of PNI on biopsy may not universally predict a poorer prognosis. Validation of our results in larger, multi‐institutional cohorts is still warranted.

## AUTHOR CONTRIBUTIONS

*Research conception and design*: Hiroshi Miyamoto. *Data analysis and interpretation*: Yuki Teramoto, Ying Wang and Hiroshi Miyamoto. *Drafting of the manuscript*: Yuki Teramoto. *Critical revision of the manuscript*: Ying Wang and Hiroshi Miyamoto. All authors have approved the final version of the manuscript.

## CONFLICT OF INTEREST STATEMENT

The authors confirm that there are no conflicts of interest to declare.

## Supporting information


**Fig. S1.** Prognostic significance of the absence vs. presence of PNI on biopsy. Kaplan–Meier curves for BCR‐free survival in the entire cohort of patients (A), as well as in those with biopsy GG1 (B), GG2 (C), GG3 (D), GG4 (E), GG5 (F) or GG4–5 (G) cancer, without vs. with PNI. Comparison between 2 groups was made by the log‐rank test. Bx, biopsy.

**Fig. S2.** Prognostic significance of the number of PNI sites on biopsy. Kaplan–Meier curves for BCR‐free survival in the entire cohort of patients where PNI was detected in 0, 1, 2, 3, 4 or 5–6 biopsy sites (A) or 0, 1 or 2–6 biopsy sites (B), as well as in those with biopsy GG4 (C) or GG5 (C) cancer where PNI was detected in 0, 1 or 2–6 biopsy sites. Comparison between 2 groups was made by the log‐rank test. Bx, biopsy.

**Fig. S3.** Prognostic significance of the number of PNI foci on biopsy. Kaplan–Meier curves for BCR‐free survival in the entire cohort of patients where PNI was detected in 0, 1, 2, 3, 4 or 5–10 foci (A) or 0, 1 or 2–10 foci (B), as well as in those with GG4 (C) or GG5 (D) cancer where PNI was detected in 0, 1 or 2–10 foci. Comparison between 2 groups was made by the log‐rank test. Bx, biopsy.

**Fig. S4.** Prognostic significance of the status of PNI on in radical prostatectomy specimens. Kaplan–Meier curves for BCR‐free survival in the entire cohort of patients without vs. with PNI. Comparison between the 2 group was made by the log‐rank test.

**Table S1.** Clinicopathologic characteristics of the entire cohort.

**Table S2.**Clinicopathologicfeatures in patients with vs. without PNI on biopsy.

**Table S3.** Multivariable analysis of prognostic factors, including the absence vs. presence of PNI on biopsy, in the entire cohort.

**Table S4.** Multivariable analysis of prognostic factors, including the absence vs. presence of PNI on biopsy, in biopsy GG3 cases.

**Table S5.** Multivariable analysis of prognostic factors, including the absence vs. presence of PNI on biopsy, in biopsy GG4 cases.

**Table S6.**Clinicopathologicfeatures in patients with PNI in a single biopsy site vs. multiple biopsy sites.

**Table S7.** Multivariable analysis of prognostic factors, including PNI in a single biopsy site vs. multiple biopsy sites, in the entire cohort.

**Table S8.** Multivariable analysis of prognostic factors, including PNI in a single biopsy site vs. multiple biopsy sites, in biopsy GG2 cases.

**Table S9.** Multivariable analysis of prognostic factors, including PNI in a single biopsy site vs. multiple biopsy sites, in biopsy GG3 cases.

**Table S10.**Clinicopathologicfeatures in patients with PNI in a single focus vs. multiple foci on biopsy.

**Table S11.** Multivariable analysis of prognostic factors, including PNI in a single focus vs. multiple foci on biopsy, in the entire cohort.

**Table S12.** The status of PNI on biopsy versus prostatectomy.
