# Supplementary material for: Quantification of perineural invasion on prostate biopsy improves risk stratification in biopsy Grade Group 2–3 cancer
Source: BJUI Compass. 2026 Mar 31;7(4):e70196. doi: 10.1002/bco2.70196 (PMC13098363; doi:10.1002/bco2.70196)
Supplement: Supplementary file 1 — Fig. S1. Prognostic significance of the absence vs. presence of PNI on biopsy. Kaplan–Meier curves for BCR‐free survival in the entire cohort of patients (A), as well as in those with biopsy GG1 (B), GG2 (C), GG3 (D), GG4 (E), GG5 (F) or GG4–5 (G) cancer, without vs. with PNI. Comparison between 2 groups was made by the log‐rank test. Bx, biopsy. [file BCO2-7-e70196-s005.pdf]

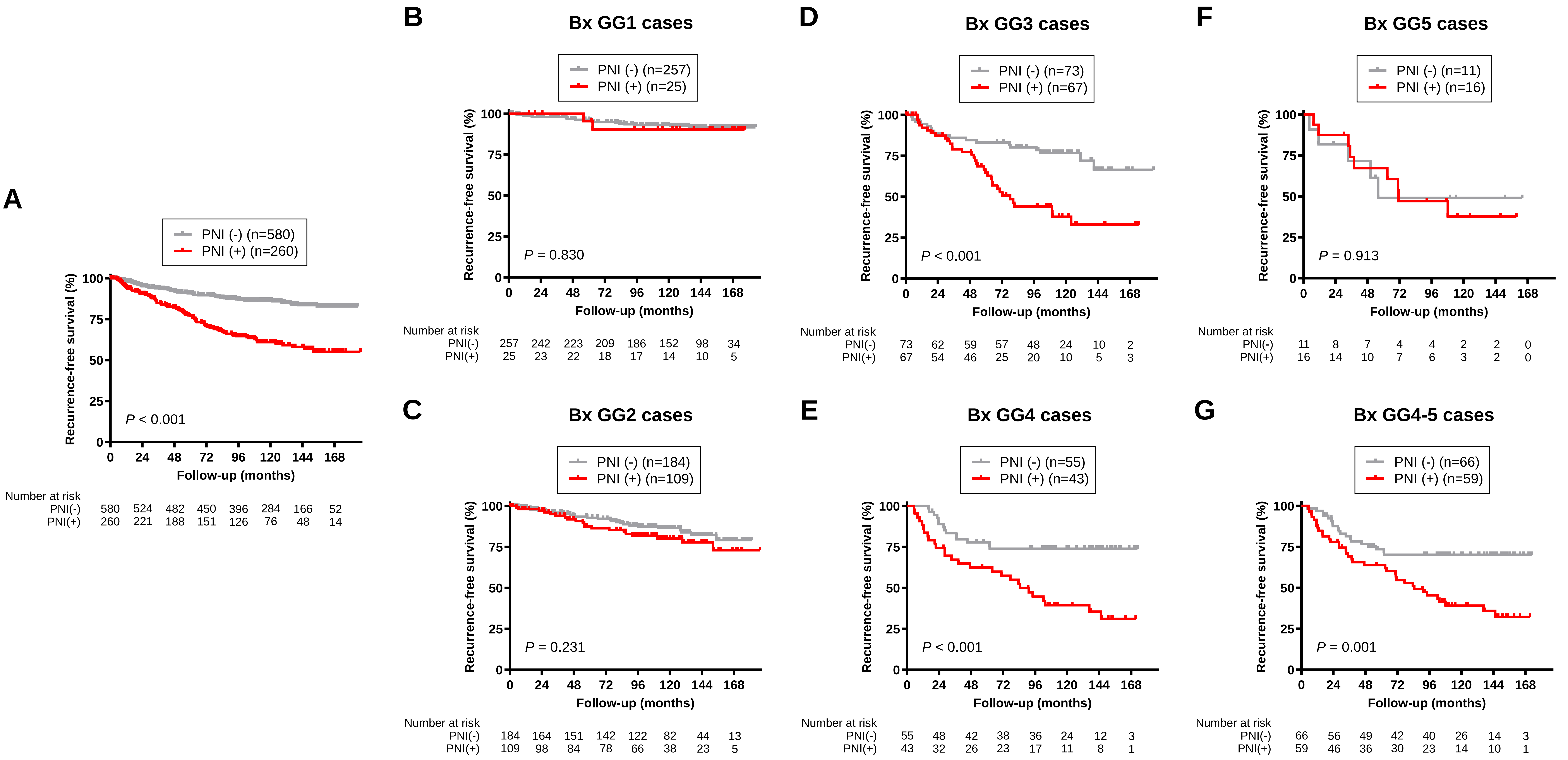

Fig. S1. Prognostic significance of the absence vs. presence of perineural invasion (PNI) on biopsy. Kaplan-Meier curves for biochemical recurrence-free survival in the entire cohort of patients (A), as well as in those with biopsy GG1 (B), GG2 (C), GG3 (D), GG4 (E), GG5 (F), or GG4-5 (G) cancer, without vs. with PNI. Comparison between 2 groups was made by the log-rank test. Bx, biopsy; GG, Grade Group.
