# Supplementary material for: Quantification of perineural invasion on prostate biopsy improves risk stratification in biopsy Grade Group 2–3 cancer
Source: BJUI Compass. 2026 Mar 31;7(4):e70196. doi: 10.1002/bco2.70196 (PMC13098363; doi:10.1002/bco2.70196)
Supplement: Supplementary file 2 — Fig. S2. Prognostic significance of the number of PNI sites on biopsy. Kaplan–Meier curves for BCR‐free survival in the entire cohort of patients where PNI was detected in 0, 1, 2, 3, 4 or 5–6 biopsy sites (A) or 0, 1 or 2–6 biopsy sites (B), as well as in those with biopsy GG4 (C) or GG5 (C) cancer where PNI was detected in 0, 1 or 2–6 biopsy sites. Comparison between 2 groups was made by the log‐rank test. Bx, biopsy. [file BCO2-7-e70196-s002.pdf]

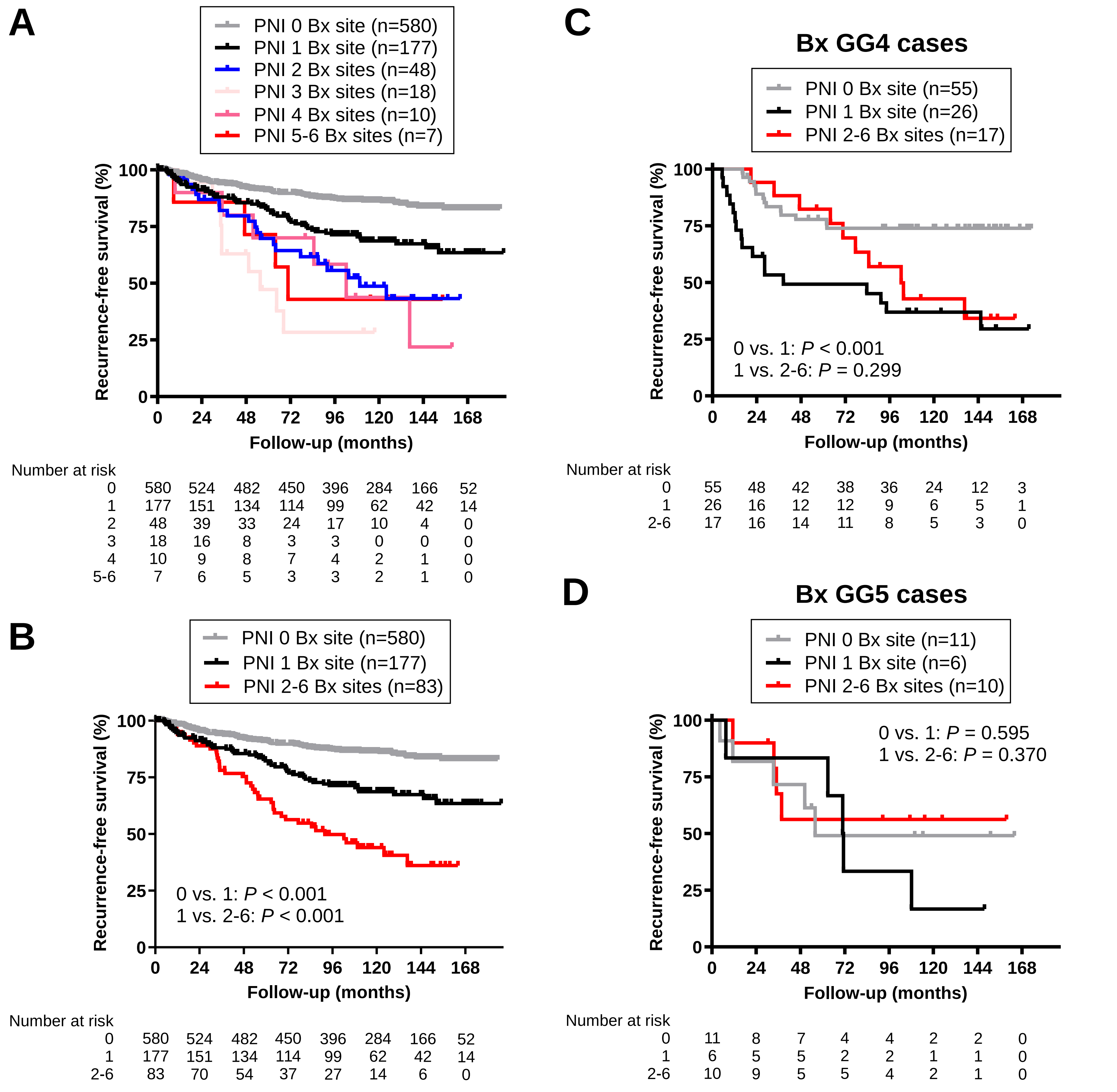

Fig. S2. Prognostic significance of the number of perineural invasion (PNI) sites on biopsy. Kaplan-Meier curves for biochemical recurrence-free survival in the entire cohort of patients where PNI was detected in 0, 1, 2, 3, 4, or 5-6 biopsy sites (A) or 0, 1, or 2-6 biopsy sites (B), as well as in those with biopsy GG4 (C) or GG5 (D) cancer where PNI was detected in 0, 1, or 2-6 biopsy sites. Comparison between 2 groups was made by the log-rank test. Bx, biopsy; GG, Grade Group.
