# Supplementary material for: Quantification of perineural invasion on prostate biopsy improves risk stratification in biopsy Grade Group 2–3 cancer
Source: BJUI Compass. 2026 Mar 31;7(4):e70196. doi: 10.1002/bco2.70196 (PMC13098363; doi:10.1002/bco2.70196)
Supplement: Supplementary file 3 — Fig. S3. Prognostic significance of the number of PNI foci on biopsy. Kaplan–Meier curves for BCR‐free survival in the entire cohort of patients where PNI was detected in 0, 1, 2, 3, 4 or 5–10 foci (A) or 0, 1 or 2–10 foci (B), as well as in those with GG4 (C) or GG5 (D) cancer where PNI was detected in 0, 1 or 2–10 foci. Comparison between 2 groups was made by the log‐rank test. Bx, biopsy. [file BCO2-7-e70196-s010.pdf]

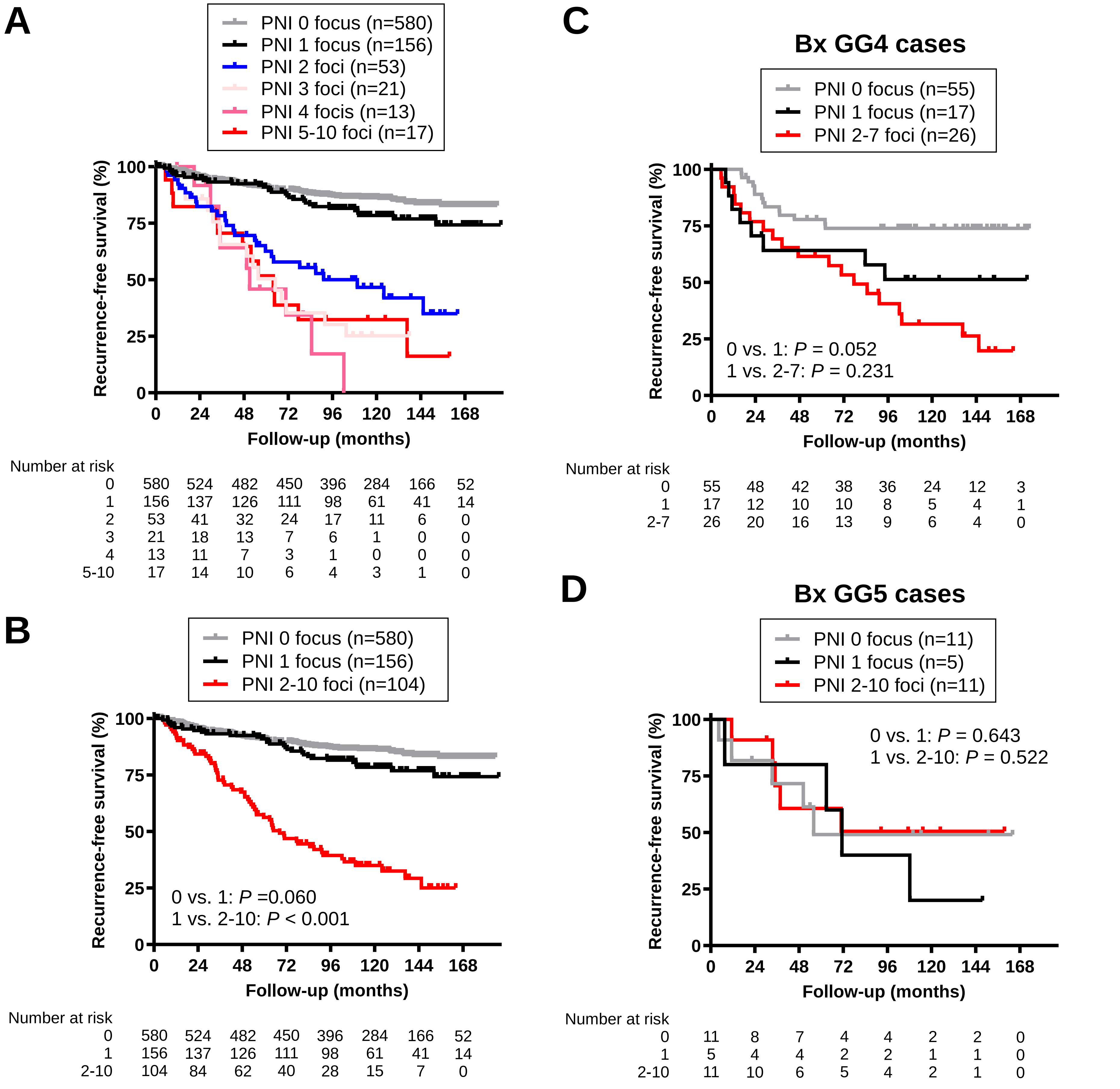


**Fig. S3.** Prognostic significance of the number of perineural invasion (PNI) foci on biopsy. Kaplan-Meier curves for biochemical recurrence-free survival in the entire cohort of patients where PNI was detected in 0, 1, 2, 3, 4, or 5-10 foci (A) or 0, 1, or 2-10 foci (B), as well as in those with biopsy GG4 (C) or GG5 (D) cancer where PNI was detected in 0, 1, or 2-10 foci. Comparison between 2 groups was made by the log-rank test. Bx, biopsy; GG, Grade Group.
