# Supplementary material for: Quantification of perineural invasion on prostate biopsy improves risk stratification in biopsy Grade Group 2–3 cancer
Source: BJUI Compass. 2026 Mar 31;7(4):e70196. doi: 10.1002/bco2.70196 (PMC13098363; doi:10.1002/bco2.70196)
Supplement: Supplementary file 4 — Fig. S4. Prognostic significance of the status of PNI on in radical prostatectomy specimens. Kaplan–Meier curves for BCR‐free survival in the entire cohort of patients without vs. with PNI. Comparison between the 2 group was made by the log‐rank test. [file BCO2-7-e70196-s013.pdf]

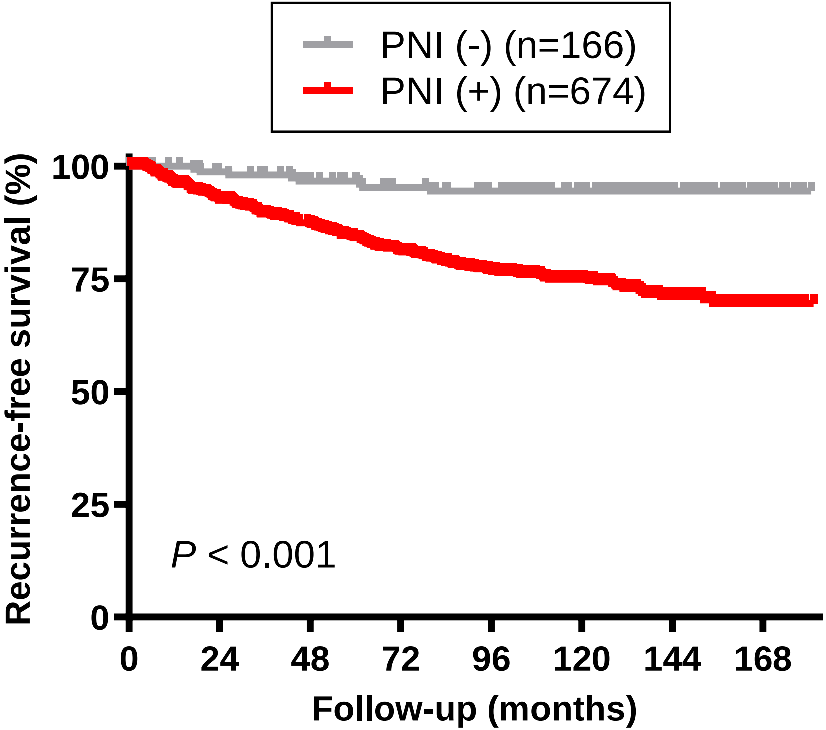

Number at risk

PNI(-)	166	154	142	132	123	91	53	20
PNI(+)	674	591	528	469	399	269	161	46

Fig. S4. Prognostic significance of the status of perineural invasion (PNI) in radical prostatectomy specimens. Kaplan-Meier curves for biochemical recurrence-free survival in the entire cohort of patients without vs. with PNI. Comparison between the 2 group was made by the log-rank test.
