# Supplementary material for: Quantification of perineural invasion on prostate biopsy improves risk stratification in biopsy Grade Group 2–3 cancer
Source: BJUI Compass. 2026 Mar 31;7(4):e70196. doi: 10.1002/bco2.70196 (PMC13098363; doi:10.1002/bco2.70196)
Supplement: Supplementary file 5 — Table S1. Clinicopathologic characteristics of the entire cohort. [file BCO2-7-e70196-s009.pdf]

Table S1. Clinicopathologic characteristics of the entire cohort.

**Age at Bx** [median (IQR) / mean ± SD, year]	63 (59-68) / 63.0 ± 6.5
**Preoperative PSA** [median (IQR) / mean ± SD, ng/mL]	6.08 (4.56-8.70) / 8.19 ± 9.53
**Bx total tumor length** [median (IQR) / mean ± SD, mm]	8.0 (2.4-17.0) / 12.9 ± 16.1
**Bx Grade Group** (highest)	
1	282 (33.6%)
2	293 (34.9%)
3	140 (16.7%)
4	98 (11.7%)
5	27 (3.2%)
**Bx PNI sites**	
0	580 (69.0%)
1	177 (21.1%)
2	48 (5.7%)
3	18 (2.1%)
4	10 (1.2%)
5	3 (0.4%)
6	4 (0.5%)
**Bx PNI foci**	
0	580 (69.0%)
1	156 (18.6%)
2	53 (6.3%)
3	21 (2.5%)
4	13 (1.5%)
5	6 (0.7%)
6	3 (0.4%)
7	4 (0.5%)
8	1 (0.1%)
9	1 (0.1%)
10	2 (0.2%)
**RP Grade Group**	
1	73 (8.7%)

2	467 (55.6%)
2 (with minor tertiary 5)	19 (2.3%)
3	151 (18.0%)
3 (with minor tertiary 5)	30 (3.6%)
4	36 (4.3%)
5	64 (7.6%)
**pT**	
2 / 2+	490 (58.3%)
3a	279 (33.2%)
3b	71 (8.5%)
**pN**	
0	643 (76.5%)
1	54 (6.4%)
X	143 (17.0%)
**Surgical margin**	
Negative	715 (85.1%)
Positive	125 (14.9%)
**RP tumor volume** [median (IQR) / mean ± SD, g]	5.4 (2.6-9.6) / 7.3 ± 7.2
**Adjuvant therapy before recurrence**	
Not performed	749 (89.2%)
Performed	91 (10.8%)
**Postoperative follow-up** [median (IQR) / mean ± SD, months]	139 (114-164) / 134.9 ± 36.5
**Biochemical recurrence**	172 (20.5%)
**Cancer-specific mortality**	14 (1.7%)

Bx, biopsy; PNI, perineural invasion; PSA, prostate-specific antigen; RP, radical prostatectomy
