# Supplementary material for: Quantification of perineural invasion on prostate biopsy improves risk stratification in biopsy Grade Group 2–3 cancer
Source: BJUI Compass. 2026 Mar 31;7(4):e70196. doi: 10.1002/bco2.70196 (PMC13098363; doi:10.1002/bco2.70196)
Supplement: Supplementary file 6 — Table S2.Clinicopathologicfeatures in patients with vs. without PNI on biopsy. [file BCO2-7-e70196-s008.pdf]

Table S2. Clinicopathologic features in patients with vs. without PNI on biopsy.

	All cases			Bx GG1 cases			Bx GG2 cases			Bx GG3 cases			Bx GG4-5 cases		
	**PNI(-)**	**PNI(+)**	*P*	**PNI(-)**	**PNI(+)**	*P*	**PNI(-)**	**PNI(+)**	*P*	**PNI(-)**	**PNI(+)**	*P*	**PNI(-)**	**PNI(+)**	*P*
n	580	260		257	25		184	109		73	67		66	59	
Age (mean ± SD; years)	63.1 ± 6.4	62.8 ± 6.7	0.530	62.3 ± 6.3	60.5 ± 7.1	0.193	62.7 ± 6.5	62.4 ± 6.7	0.702	64.9 ± 6.2	63.3 ± **6.8**	0.148	65.7 ± 5.7	64.0 ± 6.3	0.126
PSA (mean ± SD, ng/mL)	7.6 ± 6.7	9.6 ± 13.8	0.004	6.3 ± 3.4	7.5 ± 6.2	0.108	7.9 ± 7.3	7.2 ± 6.3	0.403	10.0 ± 10.6	12.8 ± 23.3	0.358	8.9 ± 8.0	11.4 ± 10.7	0.152
Bx tumor length (mean ± SD, mm)	7.2 ± 8.6	22.7 ± 20.9	<0.001	3.5 ± 4.2	12.4 ± 8.6	<0.001	9.1 ± 8.8	20.9 ± 13.5	<0.001	11.9 ± 13.0	31.0 ± 24.1	<0.001	11.2 ± 9.5	34.0 ± 26.4	<0.001
Bx GG			<0.001			NA			NA			NA			0.230
1	257 (44.3%)	25 (9.6%)		257	25		NA	NA		NA	NA		NA	NA	
2	184 (31.7%)	109 (41.9%)		NA	NA		184	109		NA	NA		NA	NA	
3	73 (12.6%)	67 (25.8%)		NA	NA		NA	NA		73	67		NA	NA	
4	55 (9.5%)	43 (16.5%)		NA	NA		NA	NA		NA	NA		55 (83.3%)	43 (72.9%)	
5	11 (1.9%)	16 (6.2%)		NA	NA		NA	NA		NA	NA		11 (16.7%)	16 (27.1%)	
Bx PNI sites			NA			NA			NA			NA			NA
1	NA	177 (68.1%)		NA	22 (88.0%)		NA	85 (78.0%)		NA	38 (56.7%)		NA	32 (54.2%)	
2	NA	48 (18.5%)		NA	3 (12.0%)		NA	15 (13.8%)		NA	17 (25.4%)		NA	13 (22.0%)	
3	NA	18 (6.9%)		NA	0 (0%)		NA	8 (7.3%)		NA	9 (13.4%)		NA	1 (1.7%)	
4	NA	10 (3.8%)		NA	0 (0%)		NA	1 (0.9%)		NA	2 (3.0%)		NA	7 (11.9%)	
5	NA	3 (1.2%)		NA	0 (0%)		NA	0 (0%)		NA	1 (1.5%)		NA	2 (3.4%)	
6	NA	4 (1.5%)		NA	0 (0%)		NA	0 (0%)		NA	0 (0%)		NA	4 (6.8%)	
Bx PNI foci			NA			NA			NA			NA			NA
1	NA	156 (60.0%)		NA	22 (88.0%)		NA	81 (74.3%)		NA	31 (46.3%)		NA	22 (37.3%)	
2	NA	53 (20.4%)		NA	3 (12.0%)		NA	16 (14.7%)		NA	19 (28.4%)		NA	15 (25.4%)	
3	NA	21 (8.1%)		NA	0 (0%)		NA	6 (5.5%)		NA	8 (11.9%)		NA	7 (11.9%)	
4	NA	13 (5.0%)		NA	0 (0%)		NA	4 (3.7%)		NA	4 (6.0%)		NA	5 (8.5%)	
5-10	NA	17 (6.5%)		NA	0 (0%)		NA	2 (1.8%)		NA	5 (7.5%)		NA	10 (16.9%)	
RP GG			<0.001			0.866			0.479			0.149			0.048
1	63 (10.9%)	4 (1.5%)		63 (24.5%)	4 (16.0%)		0 (0%)	0 (0%)		0 (0%)	0 (0%)		0 (0%)	0 (0%)	
2	362 (62.4%)	130 (50.0%)		182 (70.8%)	20 (80.0%)		144 (78.3%)	84 (77.1%)		25 (34.2%)	23 (34.3%)		11 (16.7%)	3 (5.1%)	
3	108 (18.6%)	73 (28.1%)		9 (3.5%)	1 (4.0%)		34 (18.5%)	24 (22.0%)		38 (52.1%)	26 (38.8%)		27 (40.9%)	22 (37.3%)	
4	18 (3.1%)	18 (6.9%)		0 (0%)	0 (0%)		3 (1.6%)	1 (0.9%)		3 (4.1%)	9 (13.4%)		12 (18.2%)	8 (13.6%)	
5	29 (5.0%)	35 (13.5%)		3 (1.2%)	0 (0%)		3 (1.6%)	0 (0%)		7 (9.6%)	9 (13.4%)		16 (24.2%)	26 (44.1%)	
pT			<0.001			<0.001			0.037			<0.001			<0.001
2	404 (69.7%)	86 (33.1%)		221 (86.0%)	14 (56.0%)		115 (62.5%)	52 (47.7%)		35 (47.9%)	11 (16.4%)		33 (50.0%)	9 (15.3%)	
3a	152 (26.2%)	127 (48.8%)		35 (13.6%)	11 (44.0%)		62 (33.7%)	49 (45.0%)		32 (43.8%)	39 (58.2%)		23 (34.8%)	28 (47.5%)	
3b	24 (4.1%)	47 (18.1%)		1 (0.4%)	0 (0%)		7 (3.8%)	8 (7.3%)		6 (8.2%)	17 (25.4%)		10 (15.2%)	22 (37.3%)	
pN			<0.001*			1.000*			0.549*			0.863*			0.004*
0	425 (73.3%)	218 (83.8%)		132 (51.4%)	18 (72.0%)		171 (92.9%)	104 (95.4%)		62 (84.9%)	56 (83.6%)		60 (90.9%)	40 (67.8%)	
1	23 (4.0%)	31 (11.9%)		0 (0%)	0 (0%)		7 (3.8%)	2 (1.8%)		10 (13.7%)	11 (16.4%)		6 (9.1%)	18 (30.5%)	
X	132 (22.8%)	11 (4.2%)		125 (48.6%)	7 (28.0%)		6 (3.3%)	3 (2.8%)		1 (1.4%)	0 (0%)		0 (0%)	1 (1.7%)	
Surgical margin			0.004			0.617			1.000			0.176			0.066
Negative	508 (87.6%)	207 (79.6%)		230 (89.5%)	21 (84.0%)		158 (85.9%)	93 (85.3%)		64 (87.7%)	52 (77.6%)		56 (84.8%)	41 (69.5%)	
Positive	72 (12.4%)	53 (20.4%)		27 (10.5%)	4 (16.0%)		26 (14.1%)	16 (14.7%)		9 (12.3%)	15 (22.4%)		10 (15.2%)	18 (30.5%)	
RP tumor volume (mean ± SD, g)	5.8 ± 5.2	10.7 ± 9.5	<0.001	4.2 ± 3.6	7.2 ± 4.9	<0.001	6.7 ± 5.8	8.3 ± 6.6	0.023	7.0 ± 5.6	11.1 ± 7.8	<0.001	8.3 ± 6.3	16.2 ± 13.9	<0.001
Adjuvant therapy before recurrence			<0.001			0.920			0.888			0.0497			0.076
Not performed	539 (92.9%)	210 (80.8%)		253 (98.4%)	24 (96.0%)		171 (92.9%)	101 (92.3%)		64 (87.7%)	49 (73.1%)		51 (77.3%)	36 (61.0%)	
Performed	41 (7.1%)	50 (19.2%)		4 (1.6%)	1 (4.0%)		13 (7.1%)	8 (7.3%)		9 (12.3%)	18 (26.9%)		15 (22.7%)	23 (39.0%)	

Bx, biopsy; GG, Grade Group; NA, not applicable; PNI, perineural invasion; PSA, prostate-specific antigen; RP, radical prostatectomy; SD, standard deviation

* pN0 vs. pN1.
