# Supplementary material for: Quantification of perineural invasion on prostate biopsy improves risk stratification in biopsy Grade Group 2–3 cancer
Source: BJUI Compass. 2026 Mar 31;7(4):e70196. doi: 10.1002/bco2.70196 (PMC13098363; doi:10.1002/bco2.70196)
Supplement: Supplementary file 7 — Table S3. Multivariable analysis of prognostic factors, including the absence vs. presence of PNI on biopsy, in the entire cohort. [file BCO2-7-e70196-s015.pdf]

Table S3. Multivariable analysis of prognostic factors, including the absence vs. presence of PNI on biopsy, in the entire cohort.

	HR	95% CI	*P*
**PSA**	1.005	0.994-1.018	0.373
**Biopsy tumor length**	1.000	0.991-1.009	0.995
**Biopsy Grade Group**			
1		Reference	
2	1.362	0.782-2.373	0.275
3	2.238	1.195-4.189	0.012
4	2.061	1.035-4.107	0.040
5	2.148	0.951-4.852	0.066
**PNI**			
Absence		Reference	
Presence	1.440	1.014-2.045	0.042
**Prostatectomy Grade Group**			
1-2		Reference	
3	1.572	0.997-2.478	0.051
4	2.907	1.536-5.502	0.001
5	1.898	1.052-3.426	0.033
**pT**			
2		Reference	
3a	2.842	1.850-4.364	<0.001
3b	3.733	2.114-6.591	<0.001
**Lymph node involvement**	1.910	1.149-3.177	0.013
**Surgical margin**	1.570	1.080-2.281	0.018
**Prostatectomy tumor volume**	1.016	0.996-1.037	0.116

CI, confidence interval; HR, hazard ratio; PNI, perineural invasion; PSA, prostate-specific antigen
