# Supplementary material for: Quantification of perineural invasion on prostate biopsy improves risk stratification in biopsy Grade Group 2–3 cancer
Source: BJUI Compass. 2026 Mar 31;7(4):e70196. doi: 10.1002/bco2.70196 (PMC13098363; doi:10.1002/bco2.70196)
Supplement: Supplementary file 8 — Table S4. Multivariable analysis of prognostic factors, including the absence vs. presence of PNI on biopsy, in biopsy GG3 cases. [file BCO2-7-e70196-s004.pdf]

Table S4. Multivariable analysis of prognostic factors, including the absence vs. presence of PNI on biopsy, in biopsy GG3 cases.

	HR	95% CI	*P*
**PSA**	1.011	0.997-1.026	0.134
**Biopsy tumor length**	1.008	0.991-1.025	0.354
**PNI**			
Absence		Reference	
Presence	1.918	0.977-3.763	0.058
**Prostatectomy Grade Group**			
1-2		Reference	
3	1.803	0.809-4.021	0.150
4	3.597	1.243-10.40	0.018
5	1.855	0.651-5.287	0.248
**pT**			
2		Reference	
3a	4.297	1.538-12.01	0.005
3b	4.422	1.260-15.52	0.020
**Lymph node involvement**	3.266	1.264-8.437	0.015
**Surgical margin**	1.010	0.480-2.123	0.980
**Prostatectomy tumor volume**	1.017	0.962-1.076	0.550

CI, confidence interval; HR, hazard ratio; PNI, perineural invasion; PSA, prostate-specific antigen
