# Supplementary material for: Quantification of perineural invasion on prostate biopsy improves risk stratification in biopsy Grade Group 2–3 cancer
Source: BJUI Compass. 2026 Mar 31;7(4):e70196. doi: 10.1002/bco2.70196 (PMC13098363; doi:10.1002/bco2.70196)
Supplement: Supplementary file 9 — Table S5. Multivariable analysis of prognostic factors, including the absence vs. presence of PNI on biopsy, in biopsy GG4 cases. [file BCO2-7-e70196-s012.pdf]

Table S5. Multivariable analysis of prognostic factors, including the absence vs. presence of PNI on biopsy, in biopsy GG4 cases.

	HR	95% CI	*P*
**PSA**	1.007	0.973-1.043	0.680
**Biopsy tumor length**	1.018	0.997-1.040	0.089
**PNI**			
Absence		Reference	
Presence	2.705	1.186-6.169	0.018
**Prostatectomy Grade Group**			
1-2		Reference	
3	1.214	0.250-5.891	0.810
4	1.927	0.382-9.719	0.427
5	1.318	0.241-7.220	0.750
**pT**			
2		Reference	
3a	2.318	0.884-6.082	0.087
3b	7.115	2.348-21.56	<0.001
**Lymph node involvement**	1.923	0.726-5.096	0.189
**Surgical margin**	1.962	0.762-5.055	0.163
**Prostatectomy tumor volume**	1.035	0.999-1.072	0.055

CI, confidence interval; HR, hazard ratio; PNI, perineural invasion; PSA, prostate-specific antigen
