# Supplementary material for: Quantification of perineural invasion on prostate biopsy improves risk stratification in biopsy Grade Group 2–3 cancer
Source: BJUI Compass. 2026 Mar 31;7(4):e70196. doi: 10.1002/bco2.70196 (PMC13098363; doi:10.1002/bco2.70196)
Supplement: Supplementary file 10 — Table S6.Clinicopathologicfeatures in patients with PNI in a single biopsy site vs. multiple biopsy sites. [file BCO2-7-e70196-s016.pdf]

Table S6. Clinicopathologic features in patients with PNI in a single biopsy site vs. multiple biopsy sites.

	All cases			Bx GG1 cases			Bx GG2 cases			Bx GG3 cases			Bx GG4-5 cases		
	PNI 1 Bx site	PNI 2-6 Bx sites	*P*	PNI 1 Bx site	PNI 2-6 Bx sites	*P*	PNI 1 Bx site	PNI 2-6 Bx sites	*P*	PNI 1 Bx site	PNI 2-6 Bx sites	*P*	PNI 1 Bx site	PNI 2-6 Bx sites	*P*
n	177	83		22	3		85	24		38	29		32	27	
Age (mean ± SD; years)	62.5 ± 6.7	63.5 ± 6.7	0.246	60.5 ± 7.6	60.7 ± 2.3	0.971	62.1 ± 7.0	63.7 ± 5.6	0.300	63.7 ± 6.3	62.9 ± **7.5**	0.640	63.6 ± 5.6	64.4 ± 7.2	0.624
PSA (mean ± SD, ng/mL)	8.5 ± 8.4	11.9 ± 21.1	0.069	7.1 ± 5.4	10.9 ± 11.3	0.318	6.6 ± 4.7	9.1 ± 9.9	0.095	11.4 ± 11.5	14.6 ± 33.2	0.582	11.2 ± 11.4	11.5 ± 10.1	0.902
Bx tumor length (mean ± SD, mm)	20.4 ± 14.3	36.8 ± 27.5	<0.001	11.2 ± 7.3	21.3 ± 13.6	0.053	18.3 ± 11.7	30.2 ± 15.6	<0.001	26.8 ± 18.8	36.5 ± 29.1	0.102	24.9 ± 13.8	44.9 ± 33.2	0.003
Bx GG			<0.001			NA			NA			NA			0.200
1	22 (12.4%)	3 (3.6%)		22	3		NA	NA		NA	NA		NA	NA	
2	85 (48.0%)	24 (28.9%)		NA	NA		85	24		NA	NA		NA	NA	
3	38 (21.5%)	29 (34.9%)		NA	NA		NA	NA		38	29		NA	NA	
4	26 (14.7%)	17 (20.5%)		NA	NA		NA	NA		NA	NA		26 (81.3%)	17 (63.0%)	
5	6 (3.4%)	10 (12.0%)		NA	NA		NA	NA		NA	NA		6 (18.8%)	10 (37.0%)	
RP GG			<0.001			0.654			<0.001			0.152			0.765
1	4 (2.3%)	0 (0%)		4 (18.2%)	0 (0%)		0 (0%)	0 (0%)		0 (0%)	0 (0%)		0 (0%)	0 (0%)	
2	101 (57.1%)	29 (34.9%)		17 (77.3%)	3 (100%)		68 (80.0%)	16 (66.7%)		15 (39.5%)	8 (27.6%)		1 (3.1%)	2 (7.4%)	
3	46 (26.0%)	27 (32.5%)		1 (4.5%)	0 (0%)		16 (18.8%)	8 (33.3%)		16 (42.1%)	10 (34.5%)		13 (40.6%)	9 (33.3%)	
4	11 (6.2%)	7 (8.4%)		0 (0%)	0 (0%)		1 (1.2%)	0 (0%)		5 (13.2%)	4 (13.8%)		5 (15.6%)	3 (11.1%)	
5	15 (8.5%)	20 (24.1%)		0 (0%)	0 (0%)		0 (0%)	0 (0%)		2 (5.3%)	7 (24.1%)		13 (40.6%)	13 (48.1%)	
pT			<0.001			0.823			0.001			0.407			0.011
2	76 (42.9%)	10 (12.0%)		12 (54.5%)	2 (66.7%)		47 (55.3%)	5 (20.8%)		8 (21.1%)	3 (10.3%)		9 (28.1%)	0 (0%)	
3a	80 (45.2%)	47 (56.6%)		10 (45.5%)	1 (33.3%)		35 (41.2%)	14 (58.3%)		22 (57.9%)	17 (58.6%)		13 (40.6%)	15 (55.6%)	
3b	21 (11.9%)	26 (31.3%)		0 (0%)	0 (0%)		3 (3.5%)	5 (20.8%)		8 (21.1%)	9 (31.0%)		10 (31.3%)	12 (44.4%)	
pN			<0.001*			1.000*			0.074*			0.403*			<0.001*
0	155 (87.6%)	63 (75.9%)		15 (68.2%)	3 (100%)		82 (96.5%)	22 (91.7%)		30 (78.9%)	26 (89.7%)		28 (87.5%)	12 (44.4%)	
1	11 (6.2%)	20 (24.1%)		0 (0%)	0 (0%)		0 (0%)	2 (8.3%)		8 (21.1%)	3 (10.3%)		3 (9.4%)	15 (55.6%)	
X	11 (6.2%)	0 (0%)		7 (31.2%)	0 (0%)		3 (3.5%)	0 (0%)		0 (0%)	0 (0%)		1 (3.1%)	0 (0%)	
Surgical margin			0.030			1.000			0.522			0.076			0.888
Negative	148 (83.6%)	59 (71.1%)		19 (86.4%)	2 (66.7%)		74 (87.1%)	19 (79.2%)		33 (86.8%)	19 (65.5%)		22 (68.8%)	19 (70.4%)	
Positive	29 (16.4%)	24 (28.9%)		3 (13.6%)	1 (33.3%)		11 (12.9%)	5 (20.8%)		5 (13.2%)	10 (34.5%)		10 (31.3%)	8 (29.6%)	
RP tumor volume (mean ± SD, g)	9.7 ± 8.8	13.0 ± 10.6	0.008	7.1 ± 5.2	8.3 ± 2.2	0.702	7.6 ± 5.7	10.9 ± 9.0	0.034	10.9 ± 8.1	11.4 ± 7.5	0.809	15.4 ± 14.1	17.2 ± 13.8	0.630
Adjuvant therapy before recurrence			<0.001			0.233			0.015			0.863			0.008
Not performed	155 (87.6%)	55 (66.3%)		21 (95.5%)	3 (100%)		82 (96.5%)	19 (79.2%)		27 (71.1%)	22 (75.9%)		25 (78.1%)	11 (40.7%)	
Performed	22 (12.4%)	28 (33.7%)		1 (4.5%)	0 (0%)		3 (3.5%)	5 (20.8%)		11 (28.9%)	7 (24.1%)		7 (21.9%)	16 (59.3%)	

Bx, biopsy; GG, Grade Group; NA, not applicable; PNI, perineural invasion; PSA, prostate-specific antigen; RP, radical prostatectomy; SD, standard deviation

* pN0 vs. pN1.
