# Supplementary material for: Quantification of perineural invasion on prostate biopsy improves risk stratification in biopsy Grade Group 2–3 cancer
Source: BJUI Compass. 2026 Mar 31;7(4):e70196. doi: 10.1002/bco2.70196 (PMC13098363; doi:10.1002/bco2.70196)
Supplement: Supplementary file 11 — Table S7. Multivariable analysis of prognostic factors, including PNI in a single biopsy site vs. multiple biopsy sites, in the entire cohort. [file BCO2-7-e70196-s007.pdf]

Table S7. Multivariable analysis of prognostic factors, including PNI in a single biopsy site vs. multiple biopsy sites, in the entire cohort.

	**HR**	**95% CI**	***P***
**PSA**	1.004	0.990-1.019	0.557
**Biopsy tumor length**	1.004	0.993-1.014	0.517
**Biopsy Grade Group**			
1		Reference	
2	2.146	0.494-9.322	0.308
3	4.599	1.024-20.65	0.046
4	4.197	0.861-20.46	0.076
5	3.213	0.602-17.15	0.172
**PNI**			
1 biopsy site		Reference	
≥2 biopsy sites	1.007	0.629-1.611	0.977
**Prostatectomy Grade Group**			
1-2		Reference	
3	1.376	0.722-2.622	0.332
4	1.911	0.816-4.474	0.136
5	1.834	0.800-4.204	0.152
**pT**			
2		Reference	
3a	2.837	1.318-6.106	0.008
3b	5.503	2.242-13.51	<0.001
**Lymph node involvement**	2.042	1.074-3.885	0.030
**Surgical margin**	1.246	0.724-2.146	0.427
**Prostatectomy tumor volume**	1.025	1.001-1.049	0.045

CI, confidence interval; HR, hazard ratio; PNI, perineural invasion; PSA, prostate-specific antigen
