# Supplementary material for: Quantification of perineural invasion on prostate biopsy improves risk stratification in biopsy Grade Group 2–3 cancer
Source: BJUI Compass. 2026 Mar 31;7(4):e70196. doi: 10.1002/bco2.70196 (PMC13098363; doi:10.1002/bco2.70196)
Supplement: Supplementary file 12 — Table S8. Multivariable analysis of prognostic factors, including PNI in a single biopsy site vs. multiple biopsy sites, in biopsy GG2 cases. [file BCO2-7-e70196-s011.pdf]

Table S8. Multivariable analysis of prognostic factors, including PNI in a single biopsy site vs. multiple biopsy sites, in biopsy GG2 cases.

	**HR**	**95% CI**	***P***
**PSA**	1.064	0.915-1.237	0.423
**Biopsy tumor length**	1.017	0.978-1.057	0.403
**PNI**			
1 biopsy site		Reference	
≥2 biopsy sites	2.173	0.612-7.714	0.230
**Prostatectomy Grade Group**			
1-2		Reference	
3	1.019	0.311-3.343	0.975
4	<0.001	<0.001-Inf	0.998
5	NA	NA	NA
**pT**			
2		Reference	
3a	3.970	1.204-13.10	0.024
3b	3.088	0.331-28.85	0.323
**Lymph node involvement**	2.132	0.062-72.90	0.675
**Surgical margin**	2.954	0.999-8.740	0.050
**Prostatectomy tumor volume**	1.059	0.991-1.133	0.090

CI, confidence interval; HR, hazard ratio; NA, not available; PNI, perineural invasion; PSA, prostate-specific antigen
