# Supplementary material for: Quantification of perineural invasion on prostate biopsy improves risk stratification in biopsy Grade Group 2–3 cancer
Source: BJUI Compass. 2026 Mar 31;7(4):e70196. doi: 10.1002/bco2.70196 (PMC13098363; doi:10.1002/bco2.70196)
Supplement: Supplementary file 13 — Table S9. Multivariable analysis of prognostic factors, including PNI in a single biopsy site vs. multiple biopsy sites, in biopsy GG3 cases. [file BCO2-7-e70196-s003.pdf]

Table S9. Multivariable analysis of prognostic factors, including PNI in a single biopsy site vs. multiple biopsy sites, in biopsy GG3 cases.

	**HR**	**95% CI**	***P***
**PSA**	1.011	0.996-1.027	0.157
**Biopsy tumor length**	1.005	0.986-1.024	0.636
**PNI**			
1 biopsy site		Reference	
≥2 biopsy sites	1.470	0.665-3.251	0.341
**Prostatectomy Grade Group**			
1-2		Reference	
3	2.418	0.896-6.524	0.081
4	3.161	0.806-12.39	0.099
5	2.845	0.779-10.38	0.114
**pT**			
2		Reference	
3a	4.089	0.523-31.98	0.180
3b	4.158	0.462-37.41	0.204
**Lymph node involvement**	2.326	0.645-8.386	0.197
**Surgical margin**	1.335	0.487-3.659	0.574
**Prostatectomy tumor volume**	1.022	0.956-1.091	0.525

CI, confidence interval; HR, hazard ratio; PNI, perineural invasion; PSA, prostate-specific antigen
