# Supplementary material for: Quantification of perineural invasion on prostate biopsy improves risk stratification in biopsy Grade Group 2–3 cancer
Source: BJUI Compass. 2026 Mar 31;7(4):e70196. doi: 10.1002/bco2.70196 (PMC13098363; doi:10.1002/bco2.70196)
Supplement: Supplementary file 14 — Table S10.Clinicopathologicfeatures in patients with PNI in a single focus vs. multiple foci on biopsy. [file BCO2-7-e70196-s014.pdf]

Table S10. Clinicopathologic features in patients with PNI in a single focus vs. multiple foci on biopsy.

	All cases			Bx GG1 cases			Bx GG2 cases			Bx GG3 cases			Bx GG4-5 cases		
	PNI 1 focus	PNI ≥2 foci	*P*	PNI 1 focus	PNI ≥2 foci	*P*	PNI 1 focus	PNI ≥2 foci	*P*	PNI 1 focus	PNI ≥2 foci	*P*	PNI 1 focus	PNI ≥2 foci	*P*
n	156	104		22	3		81	28		31	36		22	37	
Age (mean ± SD; years)	62.4 ± 6.9	63.5 ± 6.5	0.179	60.5 ± 7.6	60.7 ± 2.3	0.971	62.1 ± 7.0	63.3 ± 5.7	0.425	63.1 ± 6.4	63.5 ± **7.3**	0.839	64.1 ± 6.1	64.0 ± 6.5	0.933
PSA (mean ± SD, ng/mL)	8.3 ± 8.2	11.6 ± 19.3	0.055	7.1 ± 5.4	10.9 ± 11.3	0.318	6.7 ± 4.8	8.5 ± 9.2	0.187	11.1 ± 11.4	14.3 ± 30.2	0.581	11.2 ± 12.5	11.4 ± 9.7	0.945
Bx tumor length (mean ± SD, mm)	19.0 ± 13.2	35.7 ± 25.8	<0.001	11.2 ± 7.3	21.3 ± 13.6	0.053	18.0 ± 11.6	29.1 ± 15.3	<0.001	25.5 ± 18.2	35.7 ± 27.6	0.084	21.0 ± 10.5	41.8 ± 29.8	0.003
Bx GG			<0.001			NA			NA			NA			0.200
1	22 (14.1%)	3 (2.9%)		22	3		NA	NA		NA	NA		NA	NA	
2	81 (51.9%)	28 (26.9%)		NA	NA		81	28		NA	NA		NA	NA	
3	31 (19.9%)	36 (34.6%)		NA	NA		NA	NA		31	36		NA	NA	
4	17 (10.9%)	26 (25.0%)		NA	NA		NA	NA		NA	NA		17 (77.3%)	26 (70.3%)	
5	5 (3.2%)	11 (10.6%)		NA	NA		NA	NA		NA	NA		5 (22.7%)	11 (29.7%)	
RP GG			<0.001			0.654			0.114			0.109			0.998
1	4 (2.6%)	0 (0%)		4 (18.2%)	0 (0%)		0 (0%)	0 (0%)		0 (0%)	0 (0%)		0 (0%)	0 (0%)	
2	99 (63.5%)	31 (29.8%)		17 (77.3%)	3 (100%)		66 (81.5%)	18 (64.3%)		15 (48.4%)	8 (22.2%)		1 (4.5%)	2 (5.4%)	
3	33 (21.2%)	40 (38.5%)		1 (4.5%)	0 (0%)		14 (17.3%)	10 (35.7%)		10 (32.3%)	16 (44.4%)		8 (36.4%)	14 (37.8%)	
4	8 (5.1%)	10 (9.6%)		0 (0%)	0 (0%)		1 (1.2%)	0 (0%)		4 (12.9%)	5 (13.9%)		3 (13.6%)	5 (13.5%)	
5	12 (7.7%)	23 (22.1%)		0 (0%)	0 (0%)		0 (0%)	0 (0%)		2 (6.5%)	7 (19.4%)		10 (45.5%)	16 (43.2%)	
pT			<0.001			0.823			0.001			0.133			0.016
2	73 (46.8%)	13 (12.5%)		12 (54.5%)	2 (66.7%)		46 (56.8%)	6 (21.4%)		8 (25.8%)	3 (8.3%)		7 (31.8%)	2 (5.4%)	
3a	69 (44.2%)	58 (55.8%)		10 (45.5%)	1 (33.3%)		32 (39.5%)	17 (60.7%)		17 (54.8%)	22 (61.1%)		10 (45.5%)	18 (48.6%)	
3b	14 (9.0%)	33 (31.7%)		0 (0%)	0 (0%)		3 (3.7%)	5 (17.9%)		6 (19.4%)	11 (30.6%)		5 (22.7%)	17 (45.9%)	
pN			<0.001*			1.000*			0.105*			0.791*			0.003*
0	139 (89.1%)	79 (76.0%)		15 (68.2%)	3 (100%)		79 (97.5%)	25 (89.3%)		25 (80.6%)	31 (86.1%)		20 (90.9%)	20 (54.1%)	
1	7 (4.5%)	24 (23.1%)		0 (0%)	0 (0%)		0 (0%)	2 (7.1%)		6 (19.4%)	5 (13.9%)		1 (4.5%)	17 (45.9%)	
X	10 (6.4%)	1 (1.0%)		7 (31.2%)	0 (0%)		2 (2.5%)	1 (3.6%)		0 (0%)	0 (0%)		1 (4.5%)	0 (0%)	
Surgical margin			0.004			1.000			0.139			0.151			0.888
Negative	134 (85.9%)	73 (70.2%)		19 (86.4%)	2 (66.7%)		72 (88.9%)	21 (75.0%)		27 (87.1%)	25 (69.4%)		16 (72.7%)	25 (67.6%)	
Positive	22 (14.1%)	31 (29.8%)		3 (13.6%)	1 (33.3%)		9 (11.1%)	7 (25.0%)		4 (12.9%)	11 (30.6%)		6 (27.3%)	12 (32.4%)	
RP tumor volume (mean ± SD, g)	8.5 ± 7.0	14.0 ± 11.5	<0.001	7.1 ± 5.2	8.3 ± 2.2	0.702	7.5 ± 5.6	10.9 ± 8.7	0.016	9.8 ± 6.7	12.2 ± 8.6	0.196	12.2 ± 11.6	18.6 ± 14.7	0.085
Adjuvant therapy before recurrence			<0.001			0.233			0.004			0.920			0.252
Not performed	138 (88.5%)	72 (69.2%)		21 (95.5%)	3 (100%)		79 (97.5%)	22 (78.6%)		22 (71.0%)	27 (75.0%)		16 (72.7%)	20 (54.1%)	
Performed	18 (11.5%)	32 (30.8%)		1 (4.5%)	0 (0%)		2 (2.5%)	6 (21.4%)		9 (29.0%)	9 (25.0%)		6 (27.3%)	17 (45.9%)	

Bx, biopsy; GG, Grade Group; NA, not applicable; PNI, perineural invasion; PSA, prostate-specific antigen; RP, radical prostatectomy; SD, standard deviation

* pN0 vs. pN1.
