# Supplementary material for: Quantification of perineural invasion on prostate biopsy improves risk stratification in biopsy Grade Group 2–3 cancer
Source: BJUI Compass. 2026 Mar 31;7(4):e70196. doi: 10.1002/bco2.70196 (PMC13098363; doi:10.1002/bco2.70196)
Supplement: Supplementary file 15 — Table S11. Multivariable analysis of prognostic factors, including PNI in a single focus vs. multiple foci on biopsy, in the entire cohort. [file BCO2-7-e70196-s006.pdf]

Table S11. Multivariable analysis of prognostic factors, including PNI in a single focus vs. multiple foci on biopsy, in the entire cohort.

	HR	95% CI	*P*
**PSA**	1.005	0.991-1.020	0.454
**Biopsy tumor length**	1.007	0.997-1.018	0.169
**Biopsy Grade Group**			
1		Reference	
2	2.000	0.459-8.715	0.356
3	3.836	0.848-17.36	0.081
4	3.075	0.523-15.19	0.168
5	2.703	0.508-14.37	0.243
**PNI**			
1 focus		Reference	
≥2 foci	2.357	1.410-3.942	0.001
**Prostatectomy Grade Group**			
1-2		Reference	
3	1.126	0.583-2.174	0.724
4	1.664	0.698-3.963	0.250
5	1.848	0.816-4.188	0.141
**pT**			
2		Reference	
3a	2.193	1.009-4.764	0.047
3b	3.908	1.572-9.715	0.003
**Lymph node involvement**	2.171	1.144-4.118	0.018
**Surgical margin**	1.201	0.694-2.078	0.513
**Prostatectomy tumor volume**	1.028	1.003-1.053	0.028

CI, confidence interval; HR, hazard ratio; PNI, perineural invasion; PSA, prostate-specific antigen
