# Supplementary material for: Quantification of perineural invasion on prostate biopsy improves risk stratification in biopsy Grade Group 2–3 cancer
Source: BJUI Compass. 2026 Mar 31;7(4):e70196. doi: 10.1002/bco2.70196 (PMC13098363; doi:10.1002/bco2.70196)
Supplement: Supplementary file 16 — Table S12. The status of PNI on biopsy versus prostatectomy. [file BCO2-7-e70196-s001.pdf]

**Table S12.** The status of PNI on biopsy versus prostatectomy.

| PNI | | Prostatectomy | | *P* |
|---|---|---|---|---|
| | | No (n=166) | Yes (n=674) | |
| Biopsy | No (n=580) | 161 (19.2%) | 419 (49.9%) | <0.001 |
| | Yes (n=260) | 5 (0.6%) | 255 (30.4%) | |
